# Impact of paravertebral muscular atrophy in acute work-related low back pain: a retrospective cohort study

**DOI:** 10.1007/s00256-025-04989-0

**Published:** 2025-07-30

**Authors:** Rita Portela Resende, Inês Martinho, Alberto Vieira, Catarina Vieira, Nuno Neves, Manuel Ribeiro da Silva, Daniela Linhares

**Affiliations:** 1 Faculty of Medicineof , University of Porto, Porto, Portugal; 2 Department of Medical Imaging, CUF Porto Hospital, Porto, Portugal; 3 Department of Orthopedics, CUF Porto Hospital, 4100-180 Porto, Portugal; 4 Center for Health Technology and Services Research, CINTESIS, Porto, Portugal; 5 Departamento Medicina da Comunidade, Faculty of Medicine, Informação E Decisão Em Saúde, MEDCIDS, University of Porto, Porto, Portugal; 6 Department of Orthopedics, Unidade Local de Saúde de São João, Porto, Portugal

**Keywords:** Workers’ compensation, Low back pain, Disability, Paraspinal muscles, Muscular atrophy, Magnetic resonance imaging

## Abstract

**Objective:**

This study aims to assess whether paravertebral muscle atrophy, evaluated through MRI, is associated with longer disability periods in patients with acute work-related low back pain (LBP).

**Materials and Methods:**

This retrospective observational study reviewed cases of adult patients evaluated foracute work-related LBP who underwent lumbar MRI between January 2021 and August 2023. Exclusion criteria included pre-existing spinal disorders, major trauma, systemic conditions affecting recovery, or radicular symptoms. Disability was quantified using total temporary incapacity (TTI), absolute incapacity (AI), and follow-up (FU) durations. Paraspinal muscle morphology was assessed at the L4–L5 level using cross-sectional area (CSA) and fatty infiltration grading with Goutallier, Mercuri, and Kader scales. Associations were tested using *t*-tests, ANOVA, and chi-square, with significance set at *p* < 0.05.

**Results:**

A total of 87 patients (77.0% male; mean age, 36.4 ± 14.9 years) were included. The mean TTI was 30.2 ± 21.9 days. No significant associations were found between CSA or most morphological scores and disability duration. Only higher Goutallier scores in the multifidus muscle were weakly associated with longer follow-up (*p* = 0.037). A modest correlation was observed between psoas CSA and follow-up/TTI duration (*p* < 0.05).

**Conclusion:**

Paravertebral muscle atrophy, as assessed by MRI, does not appear to significantly influence short-term disability in acute work-related LBP. These findings suggest that muscle morphology plays a minor role in acute LBP recovery, contrasting with its known impact in chronic cases.

## Introduction

Since 1990, low back pain (LBP) has been the leading cause of years lived with disability [1]. Approximately 70% of the years lost due to disability from low back pain occur in individuals of working age (20–65 years) [2], highlighting the considerable burden on the active population. Among work-related musculoskeletal injuries, around 40% involve the lumbar spine^3^, resulting in a significant impact on both economic and healthcare systems.

Being the major cause of work limitation and/or absence [4−6], low back pain incurs significant societal costs, including expenses directly related to healthcare delivery and those associated with work absences resulting from days of absolute and/or partial disability. In the United States, workplace accidents involving the back result in 25% of all workers’ compensation provided by workers’ accident insurance [3], with an average cost of approximately $37,000 per affected worker (National Council of Compensation Insurance). Given this impact, identifying factors that influence recovery is essential to improving care strategies and mitigating long-term disability.

Paravertebral muscle atrophy is characterized by a decrease in the cross-sectional area (CSA) of these muscles, often accompanied by fatty infiltration, which further deteriorates muscle quality [7]. At the histological level, additional changes occur, including structural abnormalities such as an increase in core/targetoid fibers and “moth-eaten” fibers [8]. These changes compromise the muscles’ ability to effectively stabilize and support the spine, leading to a cascade of spinal dysfunctions. Such pathological changes are strongly associated with degenerative spinal conditions, including facet joint osteoarthritis, spondylolisthesis, and intervertebral disc narrowing [9]. However, the direction of this association remains unclear—atrophy may both precede and result from spinal degeneration, suggesting a possible bidirectional relationship between muscle quality and structural pathology. These degenerative processes collectively impair spinal stability and functionality, playing a significant role in the development and progression of low back pain.

While chronic LBP and its association with muscle atrophy have been extensively studied, acute LBP has received considerably less attention. There is already scientific evidence establishing a link between chronic LBP and morphological changes detectable through imaging, such as muscle atrophy and fatty infiltration. These alterations compromise spinal stability and contribute to prolonged recovery times [10−12]. Recent studies suggest a growing recognition that acute LBP may also involve underlying muscular changes similar to those observed in chronic cases [9, 13]. However, the association between paravertebral muscle atrophy and extended disability periods in acute LBP remains unclear.

This study aimed to determine whether MRI-derived measures of paravertebral muscle atrophy are associated with the duration of temporary work-related disability following acute occupational low back injury. We hypothesized that greater pre-existing muscle atrophy could be associated with longer recovery periods in this context.

## Materials and methods

**Fig. 1:**
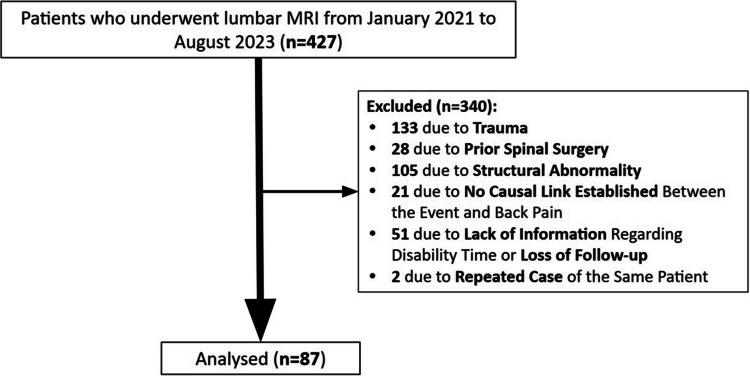
Flow diagram for the study selection process

A retrospective study was conducted, and all adult patients admitted to a central private Portuguese unit dedicated to work-related accidents were reviewed. Those who were submitted to lumbar MRI between January 2021 and August 2023 were identified. From this group, adults (> 18 years) reporting work-related acute low back pain were included. Exclusion criteria comprised a history of major trauma, relevant spinal conditions (such as spondylolisthesis, spondylolysis, or infection), concomitant disorders that could impact recovery (e.g., fibromyalgia), and non-axial symptoms such as radiculopathy (Fig. 1). Therefore, only low-energy mechanisms—such as lifting, sudden trunk rotation, or trunk flexion/extension—were included, as these are most representative of acute work-related low back pain. Cases involving major trauma were excluded, as they often result in damage to structures beyond the musculature, thus acting as confounding factors in the evaluation of paraspinal muscle alterations.

Data was collected on demographics, details on the event leading to the evaluation, and relevant clinical history. Information on dates related to this event and subsequent follow-up was also gathered, and associated disability periods were calculated.

Work-related disability was evaluated based on the duration of impairment. Absolute temporary incapacity (ATI) is the period of time, in days, during which the patient is unable to attend to work due to the condition. Partial temporary incapacity (PTI) is the number of days during which the worker is deemed capable of resuming work but is still not fully recovered, being defined somewhere between 0 and 50% (individuals with PTI higher than 50% are attributed with ATI). The overall total temporary incapacity (TTI) was calculated in days, with partial incapacities converted into fractional values (i.e., 1 day of work with 40% incapacity added as 0.4 days).

### MRI analysis

Paraspinal muscular atrophy was assessed based on MRI evaluation. The MRI analysis in this study was performed to evaluate the area and morphologic alterations in the paraspinal muscles, including the psoas, erector spinae, and multifidus [14]. Image analysis was conducted by three experienced musculoskeletal radiologists, who worked independently and were blinded to clinical data. Discrepancies were resolved by consensus. All MRI scans were acquired prior to this study for medical diagnostics and not for the purpose of this study. MR images were obtained with 1.5 T or 3 T systems (Siemens Healthcare, Erlangen, Germany), and patients were positioned supine in the MRI device. Each MRI sample contained standard T1- and T2-weighted axial sequences of the lumbar spine, standard sagittal T1- and T2-weighted sequences, sagittal STIR sequences, and standard coronal T2-weighted sequences. Synapse 3D Fujifilm’s software was used for image processing and analysis.

#### Cross-sectional area calculation

**Fig. 2:**
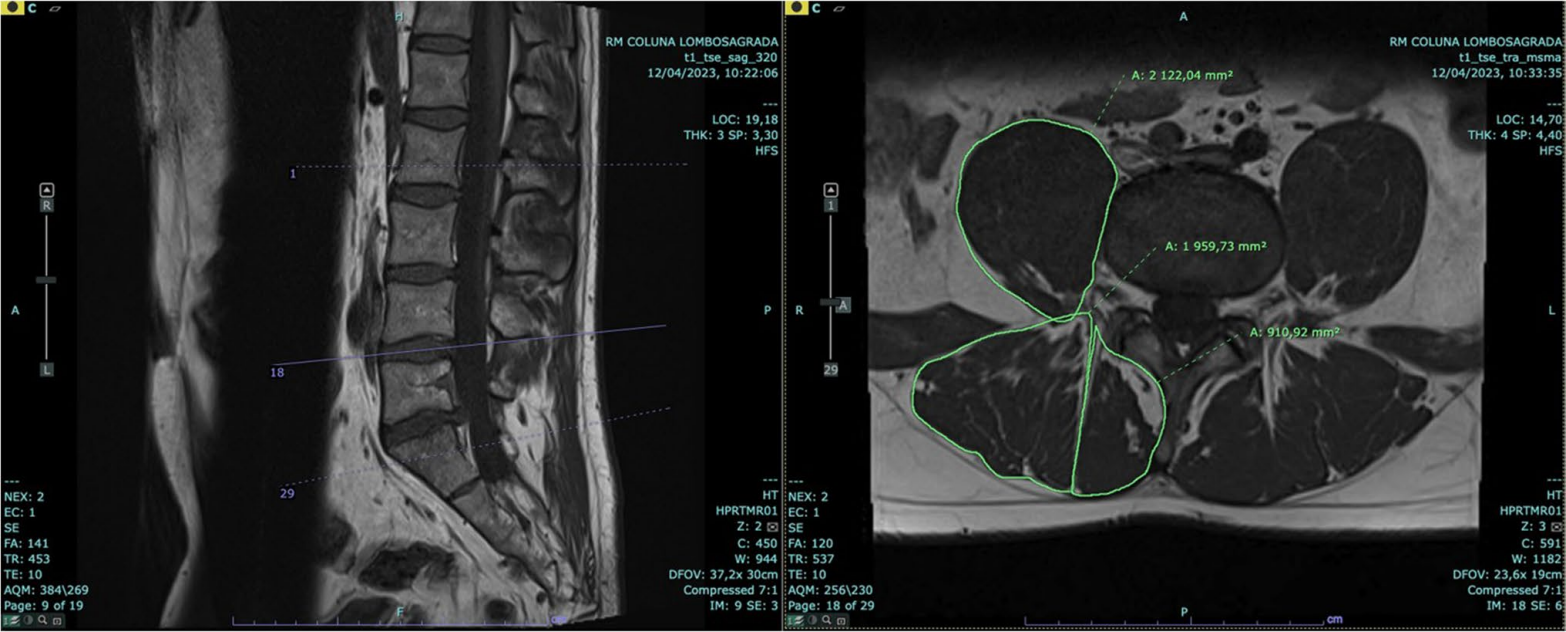
Measurement of cross-sectional area of paraspinal muscles at the L4–L5 spinal level in a patient with work-related acute low back pain

For each patient, the cross-sectional area of the psoas, erector spinae, and multifidus muscles was determined using a semiautomated software tool, allowing for precise delineation of the muscle boundaries. After the muscles were outlined manually using the software, the system automatically calculated the muscle areas based on the defined borders. All measures were conducted at the L4–L5 spinal level to ensure consistency and accuracy in comparisons (Fig. 2). This level was chosen based on the evidence from the literature demonstrating that L4–L5 is the spinal segment most affected by fatty infiltration and paraspinal muscle atrophy [15, 16], making it a key region for assessing morphological changes associated with low back pain.

#### Fatty infiltration assessment

To assess the extent of adipose infiltration and muscle atrophy, a visual grading method was used for this study. Three established semi-quantitative grading scales—Goutallier, Mercuri, and Kader—were applied to each muscle (psoas, erector spinae, and multifidus). This approach was chosen in light of the heterogeneity of grading systems used in previous studies on paravertebral muscle atrophy, with the aim of increasing comparability with existing literature and enhancing reliability [17]. Although only the Goutallier scale has been formally validated for axial musculature, the inclusion of the other two systems aimed to improve the methodological robustness of the analysis and strengthen the external validity of our findings. The images were analyzed within the predefined range from L3 to L5. The region exhibiting the maximal extent of muscle fat infiltration was selected for the grading assessment [18].

- Goutallier scale: The Goutallier scale was applied using T1-weighted images. To determine the quantity of intramuscular fat, this scale comprises five criteria: 0, no fatty infiltration; 1, fatty streaks; 2, present but less than muscle; 3, equal to muscle; and 4, greater than muscle [19].
- Mercuri scale: The Mercuri scale was also applied using T1-weighted images. To evaluate the degree of adipose infiltration in the muscle tissues, this scale comprises 6 criteria: 0, normal tissue; 1, early moth-eaten appearance, scattered small areas of increased signal; 2a, late moth-eaten appearance, numerous discrete areas of increased signal with beginning confluence comprising less than 30% of the individual muscle; 2b, late moth-eaten appearance signal with beginning confluence comprising 30–60% of the volume of the individual muscle; 3, washed-out, fuzzy appearance due to confluent areas of increased signal; and 4, end-stage appearance, muscle replaced by increased density connective tissue and fat with only a rim of fascia and neurovascular structures distinguishable) [20].
- Kader scale: The Kader scale was applied using T2-weighted images. This scale grades the muscle atrophy in three levels: mild (replacement of < 10% of muscle bulk with fat/fibrous), moderate (replacement of < 50% of muscle bulk with fat/fibrous), and severe (replacement of > 50% of muscle bulk with fat/fibrous) [14].

#### Statistical analysis

Continuous variables are presented as mean (standard deviation (SD)). Categorical variables are reported as absolute numbers and percentages.

Independent *t*-tests were conducted to compare continuous variables between groups of temporary incapacity (absolute and partial). The equality of variances was assessed using Levene’s test, and appropriate *t*-test results were reported based on whether the assumption of equal variances was met.

One-way analysis of variance (ANOVA) was used to compare continuous variables across categories of muscle alterations, as classified by the Goutallier, Kader, and Mercuri scales. When significant differences were identified, post hoc analyses were performed to explore specific group comparisons further.

Categorical variables were analyzed using the chi-square test to determine associations between groups.

Effect sizes, including Cohen’s *d*, Hedges’ correction, and Glass’ delta, were calculated to estimate the magnitude of differences between groups.

The level of statistical significance was set at *p* < 0.05. All analyses were conducted using the IBM SPSS Statistics® software, version 21.0 (SPSS Inc., Chicago, IL, USA).

The study was submitted and approved by a local ethics committee.

## Results

In total, 427 records of lumbar MRI due to work-related conditions were analyzed. After applying the inclusion and exclusion criteria, 87 patients were included, with 67 (77.0%) male and a mean age of 36.40 (14.94) years (range, 19–58).

The mean follow-up was 35.64 (27.70) days, with a mean absolute work incapacity of 28.00 (21.00) days and a partial of 8.33 (14.96) days. The mean TTI was 30.21 (21.90) days. (Table 1).

**Table 1:** Demographics

	Mean (standard deviation)
Age (years)	36.40 (14.94)
Gender (*N* of male)	67 (77%)
Absolute temporary incapacity (ATI, days)	28.00 (21.00)
Partial temporary incapacity (PTI, days)	8.33 (14.96)
Total temporary incapacity (TTI, days)	30.21 (21.90)
Follow-up (days)	35.64 (27.70)
Cross-sectional area of the psoas (cm^2^)	174.40 (41.16)
Cross-sectional area of the multifidus (cm^2^)	94.58 (17.17)
Cross-sectional area of the erector spinae (cm^2^)	163.11 (39.87)

Data is shown as mean (SD) unless otherwise indicated

The mean follow-up time was longer in males (25.80 (22.16) vs 38.58 (28.65), *p* = 0.032).

The cross-sectional area of the paraspinal muscles on MRI was measured for the three main muscle groups at the L4–L5 level: erector spinae, multifidus, and psoas. The mean areas were 163.11 cm^2^ (39.87), 94.58 cm^2^ (17.17), and 174.40 cm^2^ (41.16), respectively. Mean cross-sectional areas were significantly higher in males, when compared with females, for all muscular groups (*p* < 0.05).

A Pearson correlation analysis of the relationship between cross-sectional areas and follow-up times revealed a weak but significant correlation between the psoas area and mean follow-up (*r* = 0.279, *n* = 87, *p* = 0.009) and TTI days (*r* = 0.221, *n* = 87, *p* = 0.04). No other significant correlations were found between muscular areas and other timings or age (all *p* > 0.05).

No significant association was found between Kader and Mercuri scores and age, follow-up, or disability periods (all *p* > 0.05, Table 2). In the Goutallier classification, a statistically significant difference was found in the follow-up period for the multifidus muscle (*p* = 0.037), suggesting an association between higher Goutallier scores and longer recovery durations. Also, higher psoas Goutallier scores were associated with aging (*p* < 0.001), but disability-related variables remained unaffected.

**Table 2:** Relationship between paravertebral muscle scores and incapacity and follow-up times

			ATI	TTI	Follow-up
***Psoas***	***Goutallier***	0	27 (21)	28.16 (21.55)	34.36 (27.71)
		1	30 (21)	32.41 (22.57)	37.80 (28.40)
		2	20 (14)	19.65 (14.02)	19.25 (13.55)
		3	-	-	-
		4	-	-	-
		***p***	***0.524***	***0.427***	***0.417***
	***Mercuri***	0	28 (23)	29.13 (24.85)	34.13 (30.85)
		1	29 (20)	31.32 (20.47)	37.29 (26.25)
		2a	24 (18)	24.20 (19.12)	26.00 (23.94)
		2b	-	-	-
		3	-	-	-
		4	-	-	-
		***p***	***0.821***	***0.779***	***0.689***
	***Kader***	1	28 (23)	29.13 (24.85)	34.13 (30.85)
		2	30 (20)	31.63 (20.40)	37.68 (26.15)
		3	16 (13)	16.33 (13.32)	15.33 (13.32)
		4	-	-	-
		***p***	***0.534***	***0.477***	***0.374***
***Multifidus***	***Goutallier***	0	38 (36)	41.08 (38.60)	52.20 (45.70)
		1	24 (14)	25.27 (14.45)	31.00 (18.22)
		2	18 (15)	18.51 (14.76)	23.70 (14.40)
		3	11 (11)	10.72 (10.72)	10.60 (10.60)
		4	42 (31)	33.17 (23.17)	58.75 (46.27)
		***p***	***0.130***	***0.076***	***0.037***
	***Mercuri***	0	23 (22)	25.65 (24.64)	38.27 (35.70)
		1	29 (17)	30.20 (18.13)	34.00 (26.90)
		2a	31 (23)	32.82 (23.24)	36.70 (25.90)
		2b	27 (21)	28.85 (22.02)	34.27 (28.29)
		3	-	-	-
		4	-	-	-
		***p***	***0.678***	***0.796***	***0.967***
	***Kader***	1	23 (22)	25.65 (24.64)	38.27 (35.70)
		2	28 (16)	29.66 (17.49)	33.58 (25.95)
		3	29 (22)	31.03 (22.82)	35.12 (26.48)
		4	30 (23)	32.84 (24.91)	40.13 (31.97)
		***p***	***0.832***	***0.884***	***0.936***
***Erector spinae***	***Goutallier***	0	35 (36)	37.36 (38.47)	49.00 (44.65)
		1	26 (16)	28.48 (15.21)	34.44 (19.43)
		2	27 (18)	28.73 (18.24)	32.75 (23.50)
		3	25 (15)	24.65 (14.70)	24.25 (13.96)
		4	36 (30)	39.60 (33.15)	48.80 (45.83)
		***p***	***0.729***	***0.621***	***0.296***
	***Mercuri***	0	24 (22)	26.56 (24.72)	39.18 (35.37)
		1	27 (17)	28.79 (18.76)	33.60 (28.44)
		2a	31 (23)	31.73 (23.62)	35.06 (26.39)
		2b	28 (20)	30.63 (21.16)	36.04 (27.00)
		3	-	-	-
		4	-	-	-
		***p***	***0.836***	***0.914***	***0.965***
	***Kader***	1	24 (22)	26.56 (24.72)	39.18 (35.37)
		2	27 (17)	27.93 (18.45)	32.38 (27.91)
		3	30 (22)	31.06 (22.62)	35.22 (26.24)
		4	31 (21)	33.66 (22.52)	39.10 (29.15)
		***p***	***0.817***	***0.853***	***0.907***

Values are presented as mean (SD) days

No difference was also found when comparing the mean cross-sectional areas of paraspinal muscles between two groups of temporary incapacity: short-term (< 15 days) and long-term (≥ 15 days).

## Discussion

This study is the first to examine the impact of paravertebral muscle atrophy on recovery from acute work-related low back pain, revealing no significant relationship between paraspinal muscle trophicity, assessed by MRI, and recovery duration. While prior research has predominantly focused on chronic low back pain—demonstrating associations between advanced morphological changes such as muscle atrophy and fatty infiltration with prolonged disability and poorer outcomes [9, 13, 21–23] —our results indicate that, in acute cases without underlying structural pathology, muscle condition as evaluated by MRI does not significantly influence recovery time or functional prognosis.

Although a weak association was observed between psoas cross-sectional area and follow-up duration, no significant correlations were identified with other muscular groups. While prior literature has demonstrated a relationship between paraspinal muscle atrophy and chronic spinal conditions, such as degenerative disc disease and spinal instability [9], these associations are predominantly reported in chronic contexts. In contrast, our study specifically excluded individuals with structural abnormalities, focusing on acute, low-energy injuries without underlying pathology. This methodological approach reinforces that, in the absence of chronic degenerative changes, muscular atrophy alone does not appear to significantly influence short-term recovery.

One possible explanation for the lack of a significant association between muscle atrophy and recovery in acute low back pain is that, although structural changes such as muscle atrophy and fatty infiltration can occur in the acute phase [9, 13], their impact on functional recovery may be less pronounced compared to chronic cases. In the acute setting, transient inflammatory responses, nociceptive sensitization, and biomechanical strain may be more influential in determining the duration of disability than morphological changes identifiable on imaging [9, 24].

Our study employed established classification systems—Kader [14], Goutallier [19], and Mercuri [20]—to quantify paraspinal and psoas atrophy. Notably, only higher Goutallier scores in the multifidus showed a modest association with longer recovery, again pointing to the limited influence of structural muscle changes in acute settings. This isolated association may reflect a statistical anomaly, or alternatively, point to differences in the sensitivity and specificity of each grading system. The Goutallier scale may be more responsive to subtle fatty infiltration changes observable in the acute phase, whereas the Mercuri and Kader systems might capture broader or distinct morphological patterns. This discrepancy could also result from inter-scale variability or limited agreement between systems, highlighting the challenge of comparing semi-quantitative assessments in the absence of a validated reference standard for lumbar paravertebral muscles. Overall, these findings suggest that the prognostic value of fatty infiltration grading in acute low back pain remains uncertain and should be interpreted with caution.

The sex distribution in our cohort was skewed, with 77% male participants, who also presented with significantly greater muscle cross-sectional areas. This could reflect sex-based anatomical differences but may also relate to occupational exposure, as men are often overrepresented in physically demanding jobs that predispose to acute LBP. Nonetheless, it contrasts with general epidemiological trends showing a higher overall prevalence of LBP among women [25], suggesting the importance of considering occupational roles in future research.

To our knowledge, no other study has addressed the prognostic role of muscle morphology in acute LBP within occupational health or compensation settings, making these results both original and economically significant, especially given the high burden of acute work-related LBP on healthcare systems and insurance providers worldwide.

Although paravertebral atrophy is frequently identified on imaging, our findings do not support its use as an isolated predictor of functional prognosis or work capacity in the acute post-injury phase. Further research is needed before imaging findings can be integrated into occupational or insurance decision-making frameworks.

This study has some limitations. Its retrospective design is inherently subject to biases, including selection and recall bias. There is also a potential for selection bias related to clinical referral patterns, as patients with recurrent symptoms or pre-existing muscle alterations may have been more likely to undergo imaging and be included in the cohort. In addition, individual variations in occupational demands and leisure-time physical activity were not accounted for, which may have influenced both baseline muscle condition and recovery trajectories. The follow-up period was restricted to the duration of care within workers’ compensation clinics, limiting the assessment of longer-term outcomes.

Regarding image analysis, no inter-rater reliability assessment was performed, which constrains the evaluation of consistency across radiologists. Moreover, only one of the grading systems used was originally developed for paraspinal musculature, and none have been formally validated for use in the psoas or erector spinae muscles. This lack of standardized, validated classification systems specific to lumbar musculature may have limited the precision of morphological assessment and its correlation with clinical outcomes.

Nevertheless, the use of a rigorous and standardized MRI protocol, interpreted by experienced radiologists blinded to clinical data, combined with a relatively large and well-defined sample of 87 patients, contributes to the reliability and robustness of our findings.

Looking forward, future research should explore the role of paraspinal muscle alterations in the progression from acute to chronic low back pain (LBP) and in the development of structural conditions. The impact of occupational hazards and the risk of recurrence also merit further investigation. To support these efforts, it is essential to develop and validate imaging-based classification systems specifically designed for lumbar musculature, enhancing the clinical relevance of morphologic assessments. Such advances could enable more personalized and effective interventions, ultimately improving patient outcomes and reducing the socioeconomic burden of LBP.

This study found no significant association between paraspinal muscle atrophy and recovery time from acute work-related low back pain. These findings suggest that muscle morphology plays a minor role in acute LBP recovery, contrasting with its known impact in chronic cases.

## Data Availability

The datasets generated and analyzed during the present study are not available for open access due to institutional restrictions and the need to protect patient confidentiality. However, de-identified data may be provided by the corresponding author upon reasonable request following approval by the relevant ethics committee.
